# Editorial: Advances in esophageal cancer: treatment updates and future challenges

**DOI:** 10.3389/fsurg.2026.1950194

**Published:** 2026-10-28

**Authors:** Xuefeng Leng, Kazuhiro Noma, Seong Yong Park

**Affiliations:** 1 Department of Thoracic Surgery, Sichuan Clinical Research Center for Cancer, Sichuan Cancer Hospital & Institute, Sichuan Cancer Center, University of Electronic Science and Technology of China, Chengdu, China; 2 Department of Gastroenterological Surgery, Okayama University Graduate School of Medicine, Dentistry and Pharmaceutical Sciences, Okayama, Japan; 3 Department of Thoracic and Cardiovascular Surgery, Samsung Medical Center, Sungkyunkwan University School of Medicine, Seoul, Republic of Korea

**Keywords:** esophageal cancer, esophageal squamous cell carcinoma, esophagectomy, immunotherapy, multimodal treatment

## Introduction

Esophageal cancer remains a major global health challenge, with substantial geographical variation in incidence, histological subtype, and outcome (1). Esophageal squamous cell carcinoma (ESCC) predominates in East Asia and several other high-incidence regions, whereas adenocarcinoma constitutes an increasing proportion of cases in many Western countries. Despite advances in detection and treatment, many patients still present with locally advanced disease, for which durable control usually requires coordinated multimodal care. Neoadjuvant chemoradiotherapy followed by surgery has established survival benefits in appropriate patients (2, 3), and adjuvant nivolumab has changed postoperative management for patients with residual pathological disease after neoadjuvant chemoradiotherapy and resection (4). At the same time, the rapid development of immune checkpoint inhibitors has introduced new opportunities and new uncertainties regarding the optimal neoadjuvant regimen, the continuing role of radiotherapy, postoperative treatment selection, and the possibility of treatment escalation or de-escalation (5–7).

These changes have occurred alongside refinements in minimally invasive and robot-assisted esophagectomy, lymphadenectomy, perioperative risk assessment, nutritional support, enhanced recovery, and so on. Consequently, contemporary esophageal cancer care can no longer be defined by a single modality or a single endpoint. The challenge is to integrate oncological efficacy with surgical safety, functional recovery, quality of life, and the biological heterogeneity of both the tumor and the patient.

Against this background, we organized the Research Topic “*Advances in Esophageal Cancer: Treatment Updates and Future Challenges*,” bringing together perspectives from China, Japan, the Republic of Korea, and the wider international community. Its original scope encompassed neoadjuvant immunotherapy, whole-course management, subsequent treatment selection, contemporary surgery, and organ preservation. The resulting Research Topic depicts a field moving from uniform treatment intensification toward adaptive, risk-stratified, multidisciplinary, and patient-centered care.

### Overview and global reach of the research topic

The Research Topic includes 39 articles published across six Frontiers journals: 26 original research reports, 3 systematic reviews, 2 reviews, 2 mini reviews, 5 case reports, and one study protocol. The contributions span epidemiology and special populations, perioperative and definitive multimodal treatment, surgical strategy, biomarkers and prediction models, postoperative recovery, patient-reported outcomes, and complex endoscopic management. As of July 14, 2026, the collection had accumulated 81,421 views, comprising 3,615 Topic-page views and 77,806 article views, together with 20,176 article downloads. The official analytics map demonstrates access from widely distributed locations across the world (Figure 1,2). These metrics indicate broad dissemination and international engagement, although they should not be interpreted as direct measures of scientific quality or clinical impact.

**Figure 1:**
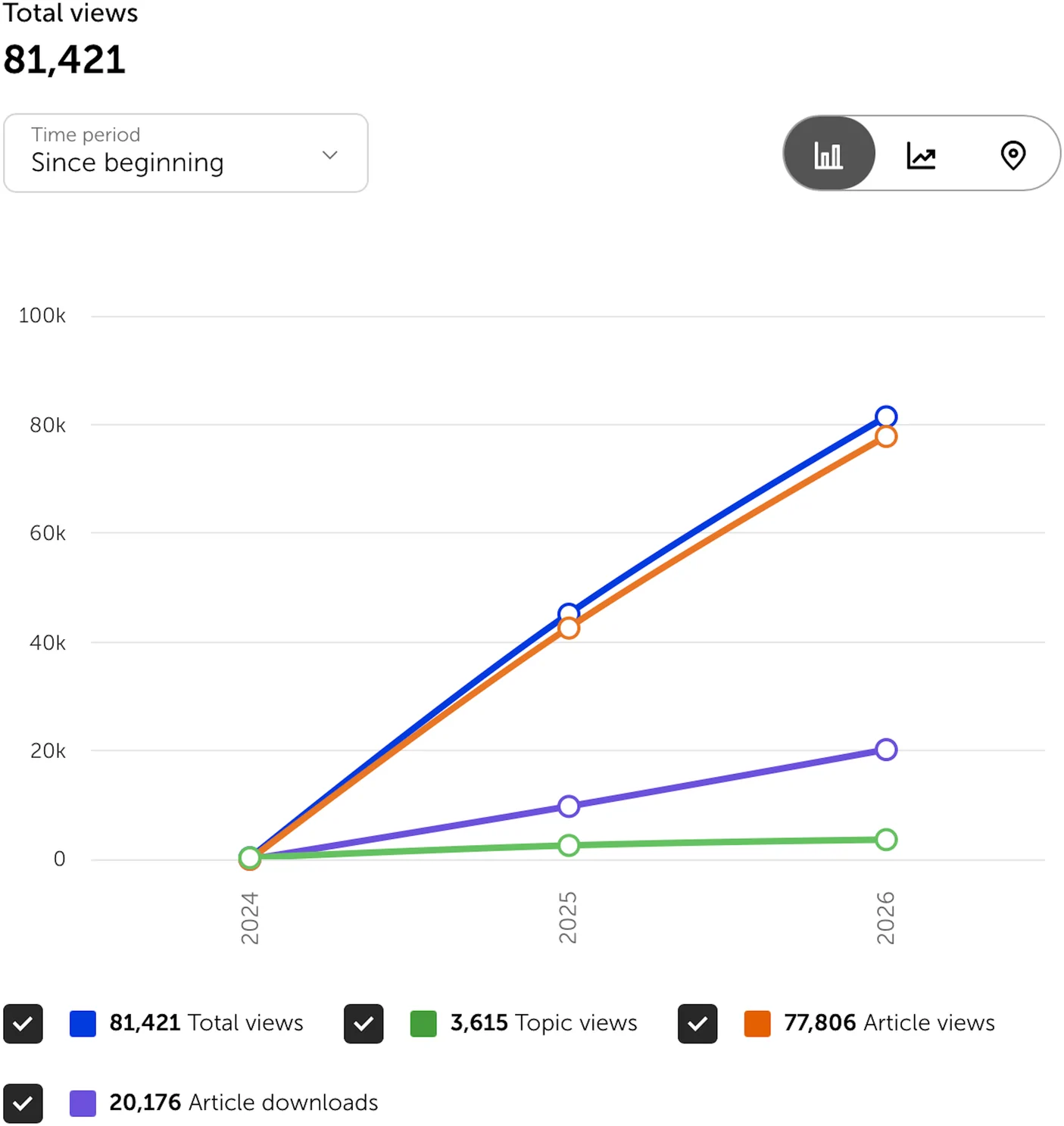
Readership and global reach of the research topic “Advances in Esophageal Cancer: Treatment Updates and Future Challenges.” readership metrics since the launch of the research topic. As of July 14, 2026, the collection had received 81,421 total views, comprising 3,615 Topic-page views and 77,806 article views, together with 20,176 article downloads.

**Figure 2:**
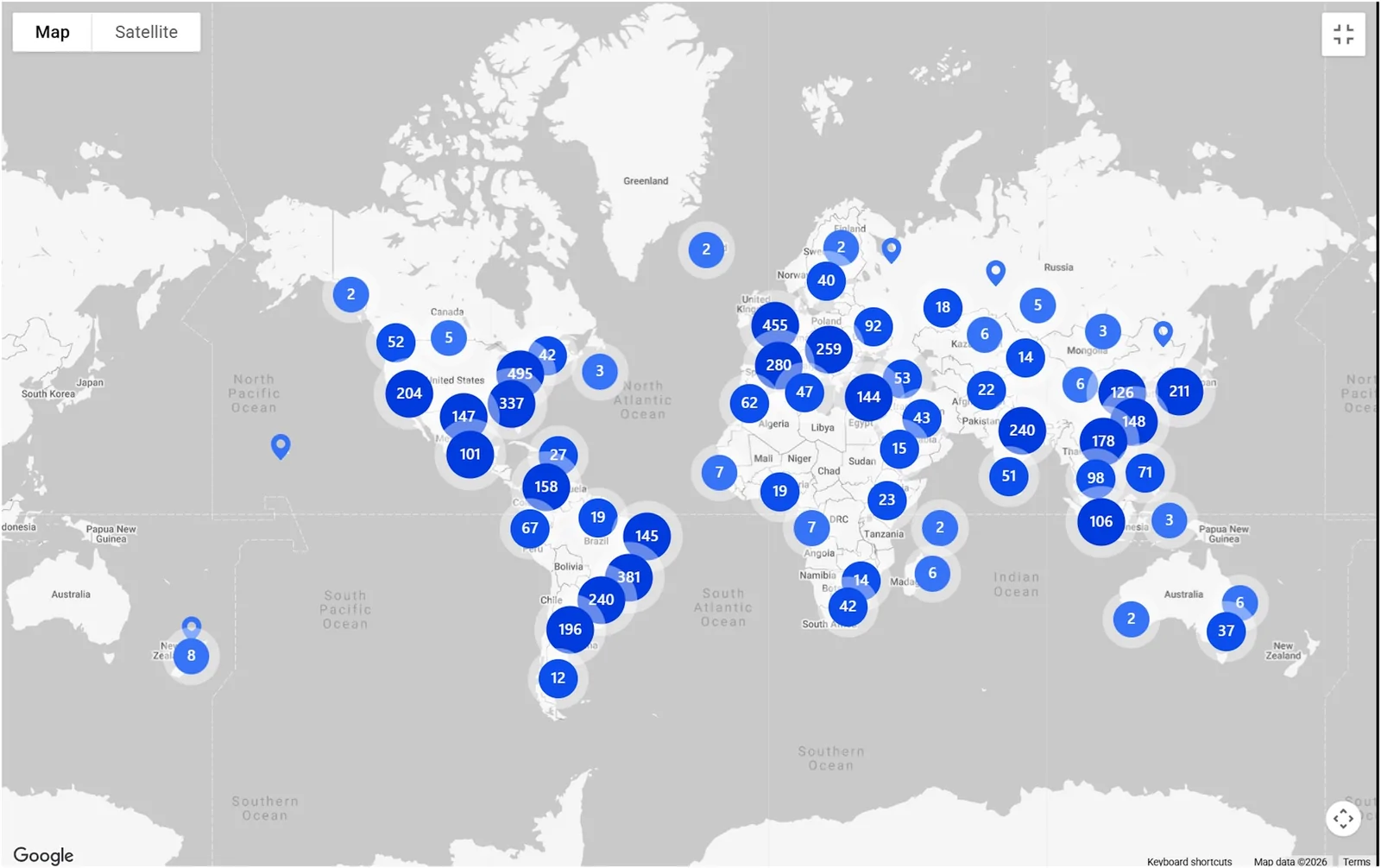
Readership and global reach of the research topic “Advances in Esophageal Cancer: Treatment Updates and Future Challenges.” geographical distribution of recorded access locations. The clustered numbers on the map represent grouped access locations rather than regional view counts. Data were obtained from the official Frontiers Research Topic analytics dashboard on July 14, 2026.

### Changing epidemiology and the challenge of special populations

Lu et al. examined global trends in esophageal cancer among adults older than 60 years and projected the burden through 2050, underscoring the growing importance of aging populations. Two complementary studies focused on heterogeneity within older surgical candidates. Li et al. explored sex-based differences in outcomes after esophagectomy, while Li et al. evaluated body mass index in relation to survival and postoperative complications. Collectively, these studies support moving beyond chronological age alone. Physiological reserve, nutritional status, body composition, comorbidity, frailty, and treatment goals should be considered together when estimating tolerance and benefit. Future trials should deliberately include older and vulnerable patients and incorporate geriatric and functional measures rather than treating age as a simple exclusion criterion.

### From treatment intensification to response-adapted multimodal therapy

The optimal perioperative regimen for locally advanced ESCC remains unsettled (6). Lin et al. reviewed the role of preoperative radiotherapy in the context of increasingly intensive chemotherapy and immunotherapy. Zhang et al. compared short-term outcomes after neoadjuvant chemoimmunotherapy, radiotherapy-containing treatment, and chemotherapy, whereas Zhao et al. compared perioperative outcomes of camrelizumab and tislelizumab based chemoimmunotherapy in a real-world cohort. These studies reflect the rapid adoption of neoadjuvant immunotherapy but also highlight a persistent distinction between pathological response and proven long-term benefit. Higher complete or major pathological response rates are clinically encouraging, yet they cannot by themselves establish superiority in event-free or overall survival.

Several contributions moved beyond a fixed-regimen framework. In patients who responded poorly to initial neoadjuvant immunochemotherapy, Lv et al. evaluated secondary induction radiotherapy before surgery, illustrating a response-adapted escalation strategy. Ding et al. described the prospective BT-NICE trial of the BTLA inhibitor tifcemalimab combined with toripalimab and chemotherapy, extending investigation beyond single-checkpoint inhibition. The more relevant future question may therefore be not whether every patient should receive chemotherapy, radiotherapy, or immunotherapy, but how these modalities should be selected, sequenced, and adapted according to early response and tumor biology.

The collection also examined settings that challenge conventional treatment boundaries. Hong et al. evaluated immunotherapy combined with definitive chemoradiotherapy for locally advanced unresectable ESCC. Zeng et al. explored neoadjuvant chemoimmunotherapy followed by surgery in ESCC with non-regional cervical lymph node metastasis, and Wang et al. assessed immunotherapy in small cell carcinoma of the esophagus. These reports suggest that systemic and local treatment integration may expand options for selected high-risk or traditionally inoperable patients. However, their retrospective or exploratory nature requires caution; selection bias and institutional expertise may substantially influence outcomes, and prospective confirmation is needed before treatment boundaries are formally redefined.

Treatment after neoadjuvant therapy and resection is another unresolved area. An et al. used nomogram-based risk stratification to examine the potential value of postoperative adjuvant therapy after neoadjuvant immunochemotherapy, while Ni et al. compared recurrence patterns between neoadjuvant and postoperative chemoradiotherapy strategies. Together, these studies reinforce the need to distinguish patients with minimal residual risk from those with persistent nodal disease, poor pathological regression, or other high-risk features. Postoperative therapy should ultimately be guided by residual disease biology and recurrence risk rather than applied uniformly.

### Refining surgery: oncological quality, technical precision, and safety

Surgery remains central for resectable disease, but its delivery continues to evolve. Peng et al. synthesized evidence comparing right- and left-sided approaches. Zhou et al. compared robot-assisted minimally invasive, video-assisted thoracoscopic, and open esophagectomy after neoadjuvant therapy, and Wei et al. examined long-term outcomes of intrathoracic versus cervical anastomosis. No platform, incision, or anastomotic location is universally optimal outside the contexts of tumor location, required lymphadenectomy, patient anatomy, surgeon experience, and institutional volume. Technical innovation should be assessed through oncological clearance, complication burden, functional recovery, learning curve, reproducibility, and resource use-not novelty alone.

The extent and interpretation of lymphadenectomy remain particularly important in ESCC. Li et al. reviewed the evolution of nodal staging from the perspective of metastatic stations, emphasizing limitations of systems based primarily on node counts. Li et al. evaluated distal perigastric lymphadenectomy for Siewert type II adenocarcinoma of the esophagogastric junction. Beyond nodal clearance, Li et al. examined long-term survival after intraoperative thoracic duct ligation, while Puccetti et al. systematically reviewed indocyanine green-assisted lymphography for preventing intraoperative chyle leak. These studies illustrate the continuing need to balance anatomical completeness with procedure related morbidity.

Rare technical hazards also carry practical lessons. Liu et al. highlighted arteria lusoria as a potentially consequential vascular variant during esophagectomy, and Ma et al. reported colonic hiatal hernia with obstruction after minimally invasive McKeown esophagectomy. Such cases reinforce the value of meticulous preoperative imaging, individualized anatomical planning, prevention of conduit and hiatus-related complications, and long-term surveillance beyond the immediate postoperative period.

### Biomarkers and prediction models: from association to actionability

A substantial component of the “Research Topic” addressed risk stratification. Readily available inflammatory and nutritional indices are attractive because they are inexpensive and easily repeated. Zhang et al. examined the intersection of inflammation and nutrition in predicting response to neoadjuvant immunochemotherapy. Fan et al. evaluated the incremental value of the aggregate index of systemic inflammation and the C-reactive protein-albumin-lymphocyte index for pulmonary complications after McKeown esophagectomy, and Ma et al. synthesized evidence on the prognostic value of the neutrophil-to-lymphocyte ratio in patients receiving neoadjuvant therapy. These markers may capture host inflammation, immune competence, and nutritional reserve, but their values are also influenced by infection, comorbidities, timing, and treatment-related changes.

Other studies extended biomarker research toward immunological, molecular, and pathological features. Chen et al. correlated immune-cell subsets in the tumor microenvironment and peripheral blood with immunotherapy response. Deng et al. developed a coagulation-related gene signature and explored its potential clinical implications. Xiong et al. synthesized evidence on perineural invasion as a prognostic feature after surgery. At the level of integrated clinical prediction, Zhang et al. developed a postoperative recurrence-risk model after neoadjuvant immunochemotherapy, whereas Xie et al. developed a cancer-specific survival nomogram for locally advanced unresectable disease.

Collectively, these contributions demonstrate both the promise and the current limitations of precision risk assessment. The field does not primarily need additional isolated models with favorable discrimination in development cohorts. It needs externally and prospectively validated tools that demonstrate calibration, reproducibility, clinical net benefit, and incremental value beyond conventional staging. Most importantly, a model should be judged by whether it changes a treatment decision or improves an outcome. Future strategies should integrate clinical and pathological variables with imaging, immune features, molecular data, and dynamic response measures, including circulating tumor DNA and measurable residual disease where appropriate.

### Perioperative recovery and outcomes that matter to patients

Major postoperative complications remain important determinants of recovery and long-term well-being. Hu et al. investigated risk factors for anastomotic leakage after radical esophagectomy. Chen and Li evaluated the preoperative prognostic nutritional index as a predictor of postoperative pneumonia, and Chen et al. developed a nomogram for pneumonia risk. These studies complement the inflammatory indices discussed above and support a broader perioperative framework incorporating respiratory risk reduction, nutritional optimization, prehabilitation, operative quality, and early recognition of complications.

Nutrition and symptom control are equally important. Wang et al. compared nasojejunal feeding, jejunostomy, and fine-needle catheter jejunostomy after esophagectomy, illustrating that access decisions should consider nutritional efficacy, tube-related morbidity, patient comfort, and resource use. Zhao et al. used symptom-network analysis to examine postoperative symptom interactions. This approach is valuable because fatigue, pain, sleep disturbance, eating difficulty, and psychological distress rarely occur in isolation. Survival remains fundamental, but it is no longer sufficient as the sole measure of successful care. Patient-reported outcomes, swallowing function, nutritional recovery, physical performance, return to daily activity, and quality of life should become routine endpoints in trials and clinical programs.

### Endoscopic rescue, palliation, and uncommon clinical challenges

The collection also captures less common but clinically consequential scenarios. Wang et al. reported malignant transformation along a leiomyoma–leiomyosarcoma sequence treated by endoscopic submucosal dissection, while Cai et al. described unicentric hyaline-vascular Castleman disease mimicking esophageal carcinoma. These cases emphasize diagnostic humility and the importance of pathological confirmation when a lesion does not follow an expected pattern. In complex benign or malignant strictures and fistulas, Zhang et al. reported use of a lumen-apposing metal stent for esophageal stricture with an esophagobronchial fistula, and Li et al. described fully covered stenting for a very high malignant esophagotracheal fistula. Such interventions illustrate the role of multidisciplinary endoscopic rescue in restoring oral intake, controlling aspiration, and improving quality of life. Palliation should not be regarded as treatment failure; it is an essential component of whole-course care.

### Future challenges and priorities

Several priorities emerge from this Research Topic. First, comparative evidence must catch up with the speed of therapeutic adoption. Randomized trials should directly compare contemporary neoadjuvant strategies using event-free and overall survival, standardized surgical morbidity, and patient-reported outcomes—not pathological response alone. Histology-specific analyses are essential, because evidence generated in ESCC cannot automatically be extrapolated to adenocarcinoma. Second, multimodal therapy should evolve toward response adaptation. Early imaging and endoscopic response, pathological regression, circulating biomarkers, and measurable residual disease may eventually guide escalation for resistant disease and de-escalation for highly responsive disease.

Third, the postoperative setting requires sharper biological selection. Patients with residual nodal disease, poor regression, or molecular evidence of persistence may merit intensified treatment, whereas those with very low residual risk may be spared unnecessary toxicity. Fourth, organ preservation remains a major gap. Although it was a central objective of the original call, few contributions directly evaluated active surveillance or definitive organ-preserving pathways. Future work must establish how clinical complete response can be assessed reliably, which patients can avoid immediate surgery, how surveillance should be performed, and whether salvage surgery can retain acceptable safety and oncological efficacy. Organ preservation should be evaluated through survival, swallowing, quality of life, and the burden of repeated assessment—not avoidance of surgery alone.

Fifth, surgical and perioperative research requires greater standardization. Definitions of lymph-node fields, anastomotic leakage, pulmonary complications, chyle leak, recovery, and quality of life should be harmonized to enable meaningful comparison across centers. Surgical innovation should be accompanied by structured quality assurance and transparent reporting of experience and learning curves. Finally, global generalizability must improve. Much of the clinical evidence in this collection arises from East Asian ESCC populations, appropriately reflecting the regional burden and research activity. International collaboration is nevertheless required to validate findings across adenocarcinoma-predominant populations, different health systems, and varied levels of access to surgery, radiotherapy, immunotherapy, nutrition, and surveillance.

## Conclusion

This Research Topic demonstrates substantial progress across the full continuum of esophageal cancer care, from population burden and treatment selection to surgery, prediction, recovery, and palliation. It also reveals that many clinically important questions remain unresolved. The future will depend less on identifying one universally superior treatment and more on determining the appropriate combination, sequence, and intensity of therapy for each patient at each stage of the disease trajectory. In practical terms, the field must move from more treatment to smarter treatment, from technical success to meaningful recovery, and from prognostic association to clinically actionable precision. Achieving this transition will require prospective evidence, standardized outcomes, international collaboration, and sustained multidisciplinary engagement centered on what matters most to patients.
